# Unlocking AI Chatbot Potential in Healthcare: Trust-Enhanced DeLone & McLean IS Success Model

**DOI:** 10.3390/healthcare14101324

**Published:** 2026-05-13

**Authors:** Mohammad Y. Sarhan, Mohammed Alarify, Mohammed Khojah

**Affiliations:** 1 Department of Management Information Systems, King Abdulaziz University, Jeddah 21589, Saudi Arabia; mysarhan@kau.edu.sa; 2 Engineering Department, Real Estate General Authority, Riyadh 13325, Saudi Arabia; malarifyy@gmail.com

**Keywords:** DeLone and McLean IS success model, healthcare service, chatbot, trust, user satisfaction, net benefits, intention to use

## Abstract

**Background:** Healthcare chatbots have emerged as a promising application of artificial intelligence in healthcare, offering potential benefits in accessibility, efficiency, and patient engagement. However, despite their growing adoption, limited research has examined the factors that determine their success from the user’s perspective. **Objective:** This study aimed to evaluate the success of a health chatbot service by applying the updated DeLone and McLean Information Systems Success Model augmented with a trust construct, examining the effects of information quality, system quality, service quality, and trust on intention to use, user satisfaction, and net benefits. **Methods:** An online survey design was employed, utilizing a structured questionnaire with 28 items measuring seven constructs on a seven-point Likert scale. Data were collected electronically from residents of Saudi Arabia between July and September 2024 using convenience sampling. Eligible participants were adults aged 18 years or older who had previously used the health chatbot service. A total of 321 valid responses were obtained. Partial Least Squares Structural Equation (PLS-SEM) was conducted using SmartPLS 3.3 software for measurement and structural model analysis. **Results:** The measurement model demonstrated acceptable reliability and validity, with composite reliability values exceeding 0.90 and average variance extracted values above 0.70 for all constructs. Structural model analysis supported eight of ten hypotheses. Trust exhibited the strongest effect on intention to use (β = 0.359, *p* < 0.001), followed by system quality (β = 0.234, *p* < 0.001) and information quality (β = 0.147, *p* < 0.01). Intention to use significantly predicted user satisfaction (β = 0.620, *p* < 0.001) and net benefits (β = 0.278, *p* < 0.001). User satisfaction demonstrated a strong positive effect on net benefits (β = 0.610, *p* < 0.001). The model explained 67.6% of the variance in intention to use, 72.7% in user satisfaction, and 71.4% in net benefits. **Conclusions:** Trust emerged as the most influential factor affecting intention to use the healthcare chatbot service, underscoring its critical role in user acceptance of health chatbot services. Information quality, system quality, and service quality exerted small to moderate effects on behavioral outcomes. These findings suggest that healthcare organizations deploying chatbot services should prioritize building user trust alongside ensuring high system and information quality to maximize user satisfaction and realized net benefits.

## 1. Introduction

The contemporary era of Artificial Intelligence (AI), characterized by unprecedented advances in machine learning algorithms and large language models, has pervaded many decision-making domains [1], enabling AI systems to imitate and augment human cognitive functions in reasoning, learning, and problem solving [2,3,4]. As this technology has matured, the healthcare sector has emerged as a particularly promising domain for AI applications [5], witnessing a revolutionary advancement in complex medical reasoning and decision support [6,7]. In this vein, Chatbots, also known as conversational agents or virtual agents, represent a type of AI application designed to simulate human-like conversations with users in natural language, providing information or services in specific domains [2,3,4]. The extant literature of healthcare services broadly acknowledges the superiority of chatbot applications in healthcare services over other AI technological alternatives, particularly in reshaping healthcare service delivery models by enhancing accessibility, personalization, and efficiency [5,8,9], while simultaneously recasting patient education, interaction, and engagement with health information [10,11,12].

In the extant literature, chatbot applications in the health services domain are neither novel nor marginal; rather, they have gained ground in the existing body of literature, reflecting a sustained and evolving scholarly interest in their potential and applications [13,14,15]. Empirical studies appear to affirm the growing role of chatbots in promoting public health messages to enhance vaccine literacy and mitigate vaccine hesitancy [16,17,18,19] improving diabetic patient education [20,21], providing emotional support for patient [22,23], assisting in remote mental health monitoring [24,25,26], aiding tobacco cessation [27,28,29,30], enhancing drug compliance, and alleviating symptoms of depression and anxiety [7,31,32].

A great achievement of the mainstream research has been to convey the message that chatbots have the toolkit to assist users with various healthcare services yet remains less conclusive regarding the extent to which users find them useful for delivering context-sensitive healthcare service advice, which needs to be thoroughly evaluated [14,33,34,35]. For instance, prior studies have been grounded in adoption driven studies, predominantly informed by Technology Acceptance Model (TAM) and Unified Theory of Acceptance and Use of Technology (UTAUT) perspectives, and focuses primarily on users’ behavioral intentions [36,37,38,39,40,41,42,43,44], leaving limited explanation on how healthcare chatbots function as effective service systems for use in practice. Others have gone so far as to demonstrate benefits and utilization of AI-powered chatbots in various healthcare environments [5,35,45,46,47], but how effectively it is utilized to provide appropriate service support to the users remains an unresolved concern in the extant literature.

With a bolder move, some scholars argued that we are overwhelmed by contributions that produced a proliferation of research on users’ behavioural intentions and acceptance studies [35], we nonetheless produce little, if at all, clear understanding about how core information system attributes contribute to user satisfaction, usage, and overall system success when engaging with AI-powered healthcare services [14,48,49,50,51]. Against this backdrop, this paper penetrated the ‘black-box’ of the factors implicated in determining the success of chatbot applications in healthcare services; an area that has hitherto been overlooked by the bulk of scholars. Following a thorough analysis of the literature, this paper seeks to fill the gap by adopting the modified DeLone and McLean Information System Success Model (D&M ISS Model) [52] to examine the mechanisms through which core information system attributes drive users’ satisfaction and subsequent use, which together serve as key indicators of overall system success in healthcare chatbot services.

While the original D&M ISS model emphasizes constructs like perceived usefulness, system quality, and service quality, it largely overlooks the role of trust. However, in the context of healthcare services, omitting trust would be a critical limitation, as it has long been fundamental to healthcare delivery, underpinning users and provider relationships and shaping reliance on healthcare outcomes [53,54]. In this vein, as chatbots become increasingly embedded in healthcare workflows, establishing trust from both patients and providers is essential for adoption and meaningful use [55,56]. Prior studies articulated that trust functions as a central mechanism that introduces perceptions of safety and credibility beyond what quality dimensions alone can ensure [57,58,59]. Users may acknowledge a chatbot’s technical quality yet still require trust before engaging in repeated use or acting upon its health-related recommendations [60]. Accordingly, trust operates as a critical mechanism through which system quality dimensions translate into user satisfaction, continued use, and net benefits. On this basis, this study was undertaken to examine the underlying mechanisms that drive users’ behavioral intention and subsequent use of healthcare chatbots and poses the following question: What are the effects of information quality, system quality, service quality, and trust on use, user satisfaction, and net benefits in the context of healthcare chatbot services?

The primary objective of this study is to evaluate the success of the Healthcare Chatbot Service from the user’s perspective by using the D&M ISS Model as a guiding framework for this research [52]. Recognized as a type of IS, the healthcare chatbot service can be assessed using the D&M ISS Model [61]. Numerous studies have employed and validated the D&M ISS Model across various domains. This model has been extensively tested in the context of e-commerce systems [62], e-government systems [63,64], and technology adoption in tourism [65]. The adaptability and robustness of the D&M ISS Model have also been demonstrated in several health IT success projects in both developing and developed nations [63,66,67,68,69]. The D&M ISS Model provides a practical framework for evaluating the effectiveness of health IS [70].

The enhancement of the original model, through the addition of the service quality variable, addresses the evolving nature of IS and the consequent shifts in the definition of “success” [71]. This modification underscores the necessity of incorporating service quality when evaluating IS, as previously advocated by several scholars [72]. Consequently, the updated D&M ISS model posits that three core components—service quality, information quality, and system quality—collectively influence system utilization and user satisfaction, thereby explaining the success of the IS platform [73]. The D&M ISS Model delineates six interconnected constructs of IS success, with a particular emphasis on the quality dimensions (service quality, information quality, and system quality). These dimensions are critical as they potentially affect users’ intent to use, actual usage, and overall satisfaction. The model posits that user satisfaction derived from these quality dimensions can lead to net benefits, which in turn reinforce continued use of the information system. The next section elaborates more on these factors and posits the research hypotheses for this study.

## 2. Theoretical Background

### 2.1. Information Quality and Use

Information quality is the degree to which an information system produces outputs that are appropriate for user decision-making [74,75]. In this vein, evaluating information quality involves assessing factors such as completeness, accuracy, uniqueness, relevance, timeliness, precision, comprehensibility, conciseness, and informativeness that pertain to relationships with users’ acceptance and intention to use IS-based services [76,77]. Based on the D&M ISS Model, the extant literature has established that users are more likely to adopt and continue using a system if they perceive the information provided to be of high quality [5,52,78,79]. Therefore, in the context of this research, we argue that when users perceive the healthcare chatbot as providing accurate information on health conditions, medication interactions, or available services, they are significantly more likely to develop a positive intention to use the system. Consequently, the following hypothesis is proposed:

**H1.**  *Information quality positively impacts the intention to use healthcare chatbot services from the user’s perspective*.

### 2.2. Information Quality and User Satisfaction

Information quality, as described earlier, also affects user satisfaction [80,81,82]. Evidence from the literature asserts that when IS-based health services deliver accurate, complete, timely, and relevant information that meets users’ informational needs, users are more likely to positively engage with the services [83,84,85,86,87]. These findings underscore the critical importance of maintaining high information standards in healthcare systems to ensure higher satisfaction. For this research, we argue that when healthcare chatbot services deliver accurate, complete, timely, and relevant information that meets users’ informational needs, users will experience greater satisfaction with the service, and, therefore, we pose the following hypothesis:

**H2.**  *Information quality positively impacts user satisfaction with healthcare chatbot services from the user’s perspective*.

### 2.3. System Quality and Use

System quality is a fundamental dimension of information systems’ success that encompasses the technical and functional characteristics of the system itself, independent of the information it produces. System quality reflects attributes such as ease of use, reliability, accessibility, response time, flexibility, integration capabilities, and overall system performance. It has been shown that system quality plays a central role in shaping how users perceive and interact with a website, as their continued use often depends on how reliable, responsive, and user-friendly they find the system to be [88]. Furthermore, the literature established that usability affects acceptability, with usability emerging as a key determinant of intention for future mobile use [89].

In healthcare IS-based services, system quality has emerged as a critical factor influencing users’ decisions to adopt and continue using digital health technologies. The relationship between system quality and intention to use is grounded in the premise that when users perceive a system as technically sound, user-friendly, and reliable, they are more likely to develop favorable intentions toward its use. Furthermore, system quality attributes such as modularity, integration, and reliability significantly impact user trust and system adoption [76]. Recent empirical evidence from digital health implementations consistently validates the positive relationship between system quality and user acceptance [90,91]. Based on this, we argue that when healthcare chatbot services demonstrate high system quality, characterized by ease of use, reliability, fast response times, intuitive interfaces, and seamless integration with existing workflows, users will develop stronger intentions to adopt and continue using the service. Consequently, the following hypothesis is proposed:

**H3.**  *System quality positively impacts the intention to use healthcare chatbot services from the user’s perspective*.

### 2.4. System Quality and User Satisfaction

System quality directly influences users’ emotional and cognitive evaluations of their interaction experiences with information systems. When systems perform reliably, respond quickly, provide intuitive interfaces, and integrate seamlessly with users’ workflows, users report higher levels of satisfaction. Research tends to affirm the significant impact of system quality on user satisfaction across various types of IS, including healthcare applications [52,63,76,79,82,92,93,94,95]. Accordingly, in this research, we argue that when healthcare chatbot services exhibit high system quality—characterized by reliability, user-friendly interfaces, fast response times, intuitive conversational flows, and seamless functionality—users will experience greater satisfaction with the service, and pose the following hypothesis:

**H4.**  *System quality positively impacts user satisfaction with healthcare chatbot services from the user’s perspective*.

### 2.5. Service Quality, Use, and Satisfaction

Service quality (ServQual) refers to the quality of support and assistance that users receive from the information system association and IT support personnel, either generally or for a specific information system. It encompasses technical support, customer responsiveness, reliability of support services, and the overall quality of interactions between service providers and users [96,97,98]. The domain has been recognized in the extant literature as a significant factor influencing users’ behavioral intentions [99,100] and actual use of digital systems [101]. Empirical evidence has affirmed the relationship between service quality and user satisfaction on the grounds of users’ general judgment of an entity’s excellence or superiority of the services that arises from comparing their expectations before experiencing the service with their actual experiences [102,103,104]. In the context of this research, we advocate that when healthcare chatbot services are accompanied by high-quality customer support, responsive assistance, technical guidance, and empathetic service interactions, users will develop stronger intentions to adopt and use these services. Therefore, the following hypothesis is proposed:

**H5.**  *Service quality positively impacts the intention to use healthcare chatbot services from the user’s perspective*.

**H6.**  *Service quality positively impacts user satisfaction with healthcare chatbot services from the user’s perspective*.

### 2.6. Intention to Use and User Satisfaction

Behavioral intention to use a system reflects a user’s estimated probability of adopting and regularly using an application or service. It is defined as “an estimate of the probability that a person will use the application” [105]. In the literature, behavioral intention has been shown to have a significant effect on users’ satisfaction formed through their experiences with the system [101]. Users with stronger intentions to use the system are more likely to develop deeper, ongoing interactions with the service, which results in increased satisfaction [94,106,107,108,109]. In this vein, we maintain that when users develop and act upon a strong intention to use healthcare chatbot services, engaging regularly with the system, their satisfaction with the service increases, and therefore, we put forward the following hypothesis:

**H7.**  *Intention to use healthcare chatbot services positively impacts user satisfaction*.

### 2.7. Intention to Use and Net Benefits

Net benefits refer to the extent to which the system contributes to the success of target subjects [52,110]. This construct encompasses both tangible benefits, such as improved efficiency, cost savings, and increased productivity, and intangible benefits, including enhanced decision-making, improved quality, and increased user empowerment [111,112]. However, the measurement of net benefits should be contextualized according to the specific system and level of impact under examination [113], recognizing that user perception and satisfaction have emerged as pivotal proxies for evaluating the actual value realized from system use [110,114]. Evidence from the extant literature consistently demonstrates that user satisfaction and actual system use jointly drive the realization of net benefits, with usage intensity predicting the magnitude of realized gains [115,116]. For this research, it is posited that when users develop and act upon strong intentions to use healthcare chatbot services, demonstrated through consistent and meaningful engagement with the system, the range of user benefits derived from such use increases. These net benefits encompass improved access to reliable health information, reduced healthcare system burden, and more efficient utilization of healthcare resources. Grounded in substantial empirical evidence showing that actual system usage—driven by strong intention to use—directly contributes to the realization of net benefits across healthcare and organizational contexts, the following hypothesis is proposed:

**H8.**  *Intention to use healthcare chatbot services positively impacts net benefits*.

### 2.8. User Satisfaction and Net Benefits

User satisfaction and net benefits represent two distinct yet interconnected dimensions of information systems success, jointly reflecting the extent to which users realize the anticipated value and outcomes from system use—thereby shaping their attitudes and behavioural intentions toward continued engagement. User satisfaction is a crucial metric for measuring the impact of IS benefits, as well as a key aspect of implementing new technologies [117]. Within the D&M ISS Model, user satisfaction precedes and closely aligns with the realization of net benefits, underscoring its role as both an outcome of system quality and a predictor of the broader benefits that follow [52]. Building on this theoretical foundation and consistent with prior empirical evidence affirming that satisfaction functions as a direct predictor and motivator for sustained system engagement [52,94,116,118], this study posits that users who experience high satisfaction with healthcare chatbot services through positive interactions, perceived usefulness, and trust are more likely to realize greater individual benefits. Satisfied users tend to engage more consistently with the service, extract higher value from its functionalities, and contribute to broader system-level gains through continued use. Accordingly, the following hypothesis is proposed:

**H9.**  *User satisfaction with healthcare chatbot services positively impacts net benefits*.

### 2.9. Trust and Intention to Use

Trust represents one of the most critical psychological constructs influencing technology adoption, particularly in healthcare and online service contexts. A study shows that trust exists when one party has confidence in the reliability and integrity of an exchange partner [119]. In fact, all social interactions could collapse without trust [120]. In the context of IS-based services, maintaining high levels of trust is essential for sustaining long-term client relationships, especially as direct, one-on-one interactions diminish. Consequently, many studies over the past decade have conceptually and empirically explored factors influencing users’ trust [121]. Research has affirmed the critical importance of trust in driving intention to use healthcare and digital health technologies [80,106,122]. Based on substantial empirical evidence demonstrating that trust is a critical driver of behavioral intention to use across diverse healthcare, digital health, and technology adoption contexts, the following hypothesis is proposed:

**H10.**  *Trust positively impacts the intention to use healthcare chatbots from the user’s perspective*.

**Figure 1:**
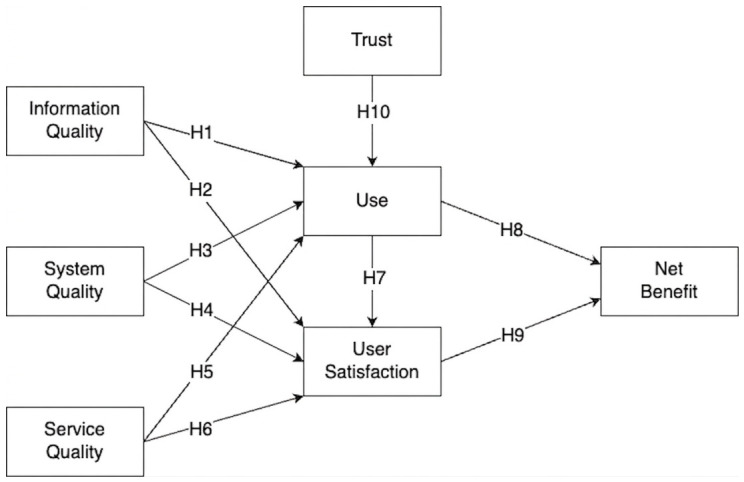
Conceptual diagram of the DeLone and McLean IS Success Model used in the study.

To sum up this section, the D&M ISS Model suggests that the success of an IS is a multidimensional construct determined by the interrelationships among information quality, system quality, and service quality, which collectively influence user satisfaction and intention to use, ultimately leading to individual and organizational net benefits. This causal framework provides a comprehensive basis for evaluating the effectiveness and user-perceived value of healthcare chatbot services, enabling a systematic understanding of how technical performance, service delivery, and user trust converge to shape overall system success. The proposed conceptual model was formulated as depicted in Figure 1 below.

## 3. Materials and Methods

A structured survey was developed to evaluate the relationships within the research model, containing indicators directly related to each construct. These indicators were adopted from pertinent literature [52,63,64]. The measurement items were tailored to specifically assess users’ experience and perceptions regarding the Ministry of Health (MOH) chatbot service within its functional context. The chatbot integrates automated responses with optional live chat support and provides key services, including locating primary healthcare and on-duty centers, managing users’ appointments, directing users to teleconsultation platforms, providing guidance on healthcare procedures (e.g., medical leave application and timelines), delivering prescription-related services, and connecting users to service representatives for inquiries, complaints, and reports.

### 3.1. Pilot Study

Prior to the main data collection, a pilot study was conducted with 10 experts who hold a master’s degree or higher. The pilot’s aim was to evaluate the clarity, reliability, and face validity of the survey items through an iterative process. Because the items were adapted from established measures in prior studies, the primary aim of the pilot was to ensure that the wording was clear, the items were understandable, and the instrument was suitable for the target participants. Feedback from the experts led to minor revisions in the item wording to improve clarity and comprehensibility. No major changes to the instrument’s structure were required.

### 3.2. Measurement Items

Each of the seven constructs in our research model was measured using four items, selected and adapted from established scales in previous studies. The precise source for each construct’s items is provided in Table 1.

All items were measured using a seven-point Likert-type scale, ranging from (1) “Strongly Disagree” to (7) “Strongly Agree”. Additionally, a demographic section was included to capture gender, age, and education level.

### 3.3. Sampling Method

We employed a non-probability sampling method, specifically convenience sampling, to recruit participants from the Kingdom of Saudi Arabia. This approach was chosen because the target population was not centrally registered and could only be reached via online platforms [123,124]. However, it may introduce selection bias and limit the extent to which the sample can be considered representative of the broader population [125,126]. The study was advertised through social media and WhatsApp groups. The inclusion criteria required respondents to be at least 18 years old, Saudi residents, and to have used at least one healthcare chatbot service in the past. Any participants who did not meet these criteria were excluded from further analysis.

### 3.4. Sample Size

The minimum recommended sample size for Partial Least Squares Structural Equation Modeling (PLS-SEM) is commonly determined using the “10-times rule,” which advises that the minimum sample should be ten times the largest number of indicators used to measure any single construct, or ten times the largest number of structural paths directed at any endogenous construct [127,128].

In our model, each construct is measured by 4 items, and our structural model includes 7 constructs and 10 hypotheses. The maximum number of predictors pointing to any construct is 6. Therefore, the minimum sample size requirement is 60. However, in most management, health informatics, and IS studies using PLS-SEM, a sample size between 200 and 300 is generally considered robust [129].

### 3.5. Data Collection

Data collection was performed via an electronic survey built with Google Forms. The survey link was distributed between July and September 2024, accompanied by an invitation message specifying the study’s purpose and inclusion criteria. The survey began with an introductory section outlining the study’s purpose and assuring participants that no personally identifiable information would be collected. Participation was entirely voluntary, with respondents able to withdraw from the survey at any time without penalties.

### 3.6. Ethical Considerations

Ethical approval for this study was waived by the Research Ethics Committee at King Abdulaziz University, which granted an exemption under Reference No. (37-25). Participation was completely voluntary, and all potential participants were informed they could withdraw at any time without providing a reason or facing any penalty. No identifying information was collected, and all survey data remained anonymous. Participants did not receive any compensation for participating in the study. The research was funded by the KAU Endowment (WAQF) at King Abdulaziz University, Jeddah, Saudi Arabia. The funder had no role in the study design, data collection, data analysis, interpretation of results, manuscript preparation, or the decision to submit the manuscript for publication.

### 3.7. Quality and Analysis

Collected responses were downloaded and labeled using IBM SPSS Statistics (Version 29) for descriptive analysis. Responses were screened for incompleteness, duplication, extreme values, and outliers. No evidence of significant bias or systematic data issues was detected in the retained responses. The data were subsequently processed using Smart PLS 3.3 software for structural equation modeling analysis. Smart PLS was selected because it is a suitable approach for examining a model with multiple latent constructs, reflective indicators, and a relatively complex structural framework. Indices and factor loading thresholds were set according to established standards [130,131,132,133]. Detailed results of the analysis are presented in the following section.

## 4. Results

This study collected responses from 321 participants (n = 321), which exceeded the minimum requirements implied by the structural complexity of the model and is considered adequate for PLS-SEM estimation. To further justify sample adequacy, a post hoc power analysis was conducted using G*Power 3 [134] for an F-test (R^2^ increase) with 10 predictors. The analysis confirmed that n = 321 provides >0.99 power to detect small effects (f^2^ = 0.02), 1.00 power for medium effects (f^2^ = 0.15), and 1.00 power for large effects (f^2^ = 0.35) at α = 0.05.

### 4.1. Demographic Data

The demographic composition revealed that 79.13% (254/321) of the respondents were male. The age distribution was as follows: 18–19 years old (24/321, 7.47%), 20–29 years old (145/321, 45.16%), 30–39 years old (69/321, 21.5%), 40–49 years old (43/321, 13.4%), 50–59 years old (23/100, 7.16%), and over 60 years old (17/321, 5.3%).

### 4.2. The Assessment of the Measurement Model

The updated guidelines for assessing the measurement model include calculating individual factor loadings, cross-loadings, Cronbach’s alpha, composite reliability, convergent validity (average variance extracted or AVE) [132], inter-construct correlations, and latent variable scores. Table 2 presents the findings of the measurement model analysis. The results indicate that the factor loading (FL) values meet the minimum required level (FL > 0.7). Composite reliability (CR) values indicate that all variables exhibit an acceptable level of reliability (CR > 0.7). Similarly, Cronbach’s alpha (α) values demonstrate that all variables maintain an acceptable level of reliability (α > 0.7). Additionally, the AVE values for all variables exceed the threshold of 0.5 (AVE > 0.5). These results confirm that the measurement model satisfies loadings, cross-loadings, reliability, and validity requirements.

The Fornell–Larcker criterion stipulates that the square root of AVE for each latent variable should be greater than the correlations with other variables [135]. Thus, discriminant validity is established for all constructs. Table 3 presents the outcomes of the Fornell–Larcker criterion.

In addition, the HTMT results indicate that most construct pairs meet the recommended threshold. However, the values between Satisfaction and Net Benefit, Use and Satisfaction, Service quality and Satisfaction, and Use and Net Benefit exceed 0.90, suggesting possible overlap among these constructs and warranting cautious interpretation. Table 4 presents the outcomes of the Heterotrait–Monotrait Ratio (HTMT).

### 4.3. Testing of the Structural Model

The structural model assessment follows the updated guidelines provided by [132]. The assessment applies the following threshold values:

- Coefficient of determination (R^2^ value): R^2^ ≥ 0.25 (weak); R^2^ ≥ 0.50 (moderate); R^2^ ≥ 0.75 (substantial).
- Path coefficients: bootstrapping (5000 bootstrap samples; 321 bootstrap cases; no sign changes) [136,137].

**Figure 2:**
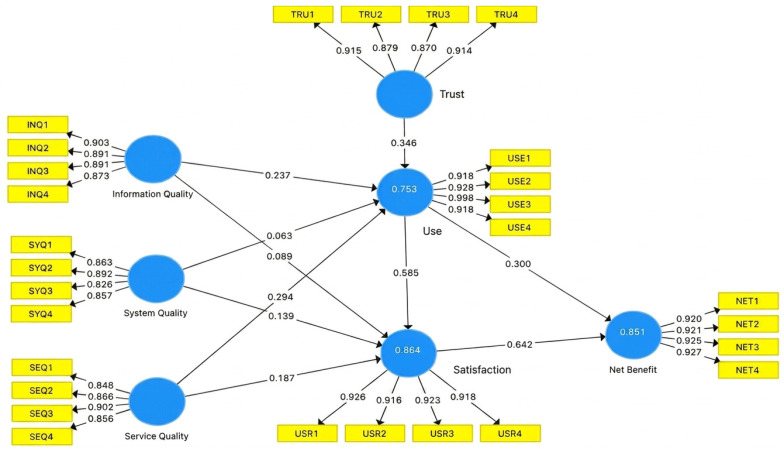
Graphical depiction of statistically validated path coefficients between model constructs.

Table 5 presents the statistical analyses of direct, total, and effect size. The path coefficient values quantify the relationships between each independent variable and the dependent variables. The analysis identified two variables with path coefficient values lower than 1.65 (*p* > 0.05), indicating non-significant relationships. Consequently, hypotheses H2 (*p* = 0.234) and H3 (*p* = 0.289) are not supported. Conversely, system quality has a significant positive impact on satisfaction (*p* = 0.018), supporting H4. Information quality has a significant positive impact on use (*p* = 0.003), supporting H1. Similarly, service quality has a significant positive impact on both satisfaction and use (*p* = 0.001), supporting H5 and H6. Finally, satisfaction has a significant positive influence on net benefits (*p* < 0.001), trust has a significant positive influence on use (*p* < 0.001), and intention to use has a significant positive influence on both net benefits and satisfaction (*p* < 0.001), supporting hypotheses H7, H8, H9, and H10. Figure 2 depicts all constructs with their respective path coefficients.

Effect size measures the impact of each predictor on the dependent construct [138]. While significance tests indicate whether a relationship exists, effect sizes convey the magnitude of these effects [127]. At the structural level, the effect of the predictor is categorized as large, medium, or small if F^2^ is 0.35, 0.15, or 0.02, respectively [139].

The R^2^ analysis revealed that the latent variables representing quality and trust explained a substantial 75% of the variance observed in user satisfaction. Additionally, the combined effects of quality and intention to use explained a high 86% of the variance in satisfaction. Finally, both intention to use and satisfaction jointly accounted for 85% of the variance in the net benefit dependent variable. These findings suggest a strong explanatory power of the model for predicting user satisfaction and net benefits.

A high Q^2^ value (>0) for endogenous variables signifies the model’s ability to explain a substantial proportion of the variance observed in these variables, exceeding the contribution of random error.

In this study, all three endogenous variables exhibited VIF values greater than 4 and tolerance values less than 0.25, indicating the potential presence of multicollinearity [140,141]. However, prior D&M ISS-based studies show that user satisfaction and net benefits are closely related, with user satisfaction often functioning as an important antecedent of net benefits [142,143,144]. Thus, the model was retained to maintain theoretical fidelity to the original framework, with the elevated VIF levels acknowledged as a limitation regarding parameter precision rather than a fundamental structural distortion [145]. Table 6 shows all the variance inflation factors.

## 5. Discussion

This study set out to examine the determinants of success for healthcare chatbot services through the lens of the D&M ISS Model. The discussion that follows interprets the empirical findings in light of the model’s theoretical propositions and articulates how the study’s findings contribute to both theoretical refinement and practical understanding of chatbot service success in healthcare—emphasizing the contextual dynamics that govern user trust, system use, and the realization of net benefits.

The demographic characteristics of the respondents are presented first to provide context for interpreting the findings. Although the sample was skewed toward male respondents, this characteristic should be considered when interpreting the findings. The results still offer useful evidence from the surveyed participants, while the gender imbalance limits the extent to which the findings can be generalized [146,147].

Drawing on SmartPLS Structural Equation Modeling (SEM), ten hypotheses were tested to evaluate the relationships among the model’s key dimensions. At the outset, the study results revealed that eight out of ten hypotheses were supported, as shown in Table 2. Among these, information quality emerged as a pivotal construct influencing users’ intention to use chatbot services. Such a finding, on one hand, underscores that enhancing the accuracy, relevance, and reliability of information provided to users significantly increases their willingness to rely on chatbot-based health services. On the other hand, this finding is in sympathy with previous findings in the mainstream literature [63,76], reaffirming that perceived information quality remains central to the success of chatbot services in the healthcare context.

While prior studies have linked increased user satisfaction to higher levels of information quality [82,85,87,111], this study diverges from that established relationship. Indeed, the findings of this study assert that information quality did not exert a significant influence on user satisfaction with healthcare chatbot services and instead indicate that it might be specific experiential factors that have become necessary for users’ satisfaction more than others that seemed to depend upon users’ perceived system quality in terms of responsiveness and chatbot fluency. This outcome aligns with [83], who similarly observed that information quality alone does not guarantee user satisfaction. It also aligns with empirical evidence from [148], who reported similar results in student information systems, where baseline informational expectations were adequately met. The lack of significance may reflect the specific characteristics of healthcare chatbot services, where users expect accurate medical information as a basic requirement rather than a differentiating factor for satisfaction. When information quality meets minimum standards of accuracy and relevance, additional improvements may yield diminishing returns on satisfaction, particularly when relational factors like trust and service quality dominate the user experience. Therefore, this study contributes to the IS success literature by highlighting that, in chatbot service applications in healthcare contexts, satisfaction may be driven less by informational quality and more by experiential qualities that shape users’ engagement with chatbot services. This underscores the need for context-specific adaptation of the D&M ISS model when applied to AI-mediated services, where technical quality dimensions may play secondary roles relative to human-like interaction capabilities.

The findings in this study throw a spotlight on the pivotal role of system quality in shaping users’ behavioral intention and satisfaction with healthcare chatbot services. Users perceive that the overall quality of the chatbot system, as reflected in its reliability, response speed, and technical stability, directly influences their willingness to engage with the technology. This outcome is in alignment with the principle that the easier the system is to use, the greater the likelihood that users will adopt and continue to use healthcare chatbot services. Here, the mainstream research is dominated by a high proportion of studies that envisage system quality as a significant predictor of both user satisfaction and intention to use, echoing the propositions of D&M ISS Model [74]. Thus, the findings of this study encapsulate the mainstream thinking and assert that enhancing system quality does not merely improve technical efficiency but also strengthens user-perceived value, ultimately fostering continued system engagement.

Contrary to expectations, system quality did not significantly influence the intention to use healthcare chatbot services. This finding highlights that providing a high-quality system alone may not be sufficient to drive the adoption of chatbots in healthcare services. While healthcare chatbots are normally developed to offer personalized services, users may prioritize other factors—such as trust and ease of use when deciding whether to continue engaging with these systems. This result is consistent with [149], who explicitly reported that system quality has no significant effect on system use in their analysis of government information systems. Similarly, ref. [150] confirmed that system quality does not significantly influence behavioral intention to use online services, suggesting that technical attributes become less salient when trust and service interactions dominate adoption decisions. The findings in this paper showed instances in which users adapted to basic system functionalities and prioritized relational factors, such as trust and service quality, over incremental improvements in technical performance. Once minimum usability thresholds are met, additional system quality enhancements may yield limited returns in terms of adoption intention, particularly in healthcare contexts where perceived reliability and human-like interaction outweigh technical sophistication. This pattern reinforces the context-dependent nature of D&M ISS model relationships and highlights trust as a critical mediator in chatbot healthcare applications.

Nevertheless, the results confirm that service quality remains an important determinant of user satisfaction, consistent with prior research. This indicates that satisfaction with healthcare chatbots depends not only on service efficiency but also on the perceived quality embedded in system interactions—elements that reinforce trust and long-term acceptance of chatbot in the healthcare environment.

Trust emerged as a strong predictor of intention to use healthcare chatbot services. The findings of this study reveal that trust exerts a strong effect on usage intention, consistent with recent systematic reviews [80] and prior IS success literature. Indeed, the absence of trust often leads to hesitation in using technology in the healthcare context, underscoring its indispensable role in sustaining continuous use. Additionally, the findings suggest that trust reinforces user satisfaction, as users who perceive chatbot interactions as credible are more likely to evaluate the service positively. This outcome indicates that fostering trust among chatbot users could enhance both their intention to use and overall satisfaction with healthcare chatbot services.

Furthermore, the results demonstrate that intention to use has a direct impact on net benefits, corroborating recent findings in the IS success literature [115,116]. Likewise, user satisfaction emerged as a strong predictor of net benefits, advocating the notion that sustained engagement with chatbot services is essential for realizing meaningful value for users. As previously emphasized by [113] that the specific benefits to be assessed are contingent upon the specific system and level of impact under examination, the finding of this study suggest that when users perceive value and satisfaction in their interactions, they are more likely to translate this experience into tangible benefits both at the individual level (e.g., service accessibility) and the organizational level (e.g., enhanced efficiency and users engagement).

As alluded to before, a great achievement of the extant studies has been the conceptualization that surmises the success of healthcare chatbot services in assisting users with various healthcare tasks. Indeed, the nature of success was taken for granted in the extant literature and somehow passed unnoticed, with no questions examining the extent to which different factors influence chatbot success. Against this backdrop, findings from this study demonstrate that the primary objective of evaluating the success of a healthcare chatbot service from the user’s perspective using the D&M ISS Model was informed by the reported results. Specifically, the result empirically indicated that chatbot success is not merely underpinned by technical quality alone, but by an integrated process in which information quality, service quality, system quality, and trust shape users’ engagement with this type of system, which in turn drives their satisfaction and perceived net benefits. Accordingly, the study provides empirical support for conceptualizing user-perceived chatbot success as a multidimensional construct encompassing quality perceptions, behavioral use, experiential satisfaction, and realized benefits.

## 6. Conclusions

This paper was concerned with the principal research question of: What are the effects of information quality, system quality, service quality, and trust on use, user satisfaction, and net benefits in the context of healthcare chatbot services? Rather than merely assuming that chatbots are inherently successful in assisting users with various healthcare tasks, this research sought to unpack the underlying mechanisms driving such success. The paper delved into the “black box” of interrelated constructs to uncover how they collectively shape user experience and perceived chatbot effectiveness in the healthcare context. By empirically examining these constructs, the study addresses a gap in the literature, where prior research has largely overlooked the interactions among these constructs in explaining chatbot success within healthcare services. Contrary to theoretical expectations, information quality significantly predicted intention to use but not user satisfaction, while system quality significantly influenced satisfaction but not intention to use. These findings indicate that accurate, relevant information primarily drives adoption decisions, whereas technical performance contributes more substantially to users’ evaluative experience of the service. Trust emerged as the strongest predictor of intention to use, underscoring its pivotal role in healthcare chatbot adoption. Collectively, these results demonstrate that information quality and system quality operate through distinct pathways in the healthcare chatbot context, with implications for the contextual adaptation of the D&M ISS Model. Moreover, both intention to use and user satisfaction were found to have a direct impact on net benefits, confirming that continued usage and satisfaction are crucial pathways through which users derive value from chatbot services. The results provided meaningful insights into the determinants of chatbot success and the dynamics shaping users’ experience when engaging with chatbot services.

### 6.1. Contribution

As the findings emerged from this research were tied back to the extant literature, the contributions of this research are now highlighted and organized into the following points. First, the present study advances the theoretical understanding of the D&M ISS Model within the context of chatbot healthcare services. The paper unearths the interrelated systems and perceptual practices—notably trust, user experience, and quality integration mechanisms—that collectively constitute the enactment of chatbot success. These practices were found to shape how users internalize chatbot effectiveness and how such perceptions translate into satisfaction, use, and ultimately, net benefits. The paper argues for nuanced changes to the extant literature that has been reluctant to treat chatbot success beyond system dimensions—such as information quality, system quality, and service quality—as discrete predictors of success. Although not dismissing their importance, the study envisages chatbot success as emerging from the dynamic interplay among quality dimensions and trust, which together mediate users’ cognitive and affective responses to system use. This reconceptualization extends the D&M ISS Model by embedding it within the socio-technical realities of healthcare, offering a more holistic explanation of how users perceive and derive value from chatbot health services.

Second, the paper offers a theoretical reframing of IS success in intelligent medical care systems—illustrating that the realization of net benefits depends not only on technical excellence but also on users’ cognitive trust, and perceived relational quality of interaction. This integrated perspective broadens the explanatory scope of the D&M ISS Model and sets a foundation for future inquiries into the success dynamics of AI-driven, trust-sensitive healthcare innovations. Such a contribution, while contextualizing D&M ISS Model application within the rapidly evolving domain of healthcare chatbot services, also serves as theoretical groundings to bridge the traditional divide between system-centric measures of success and the human-centered realities of healthcare technology adoption.

Third, the application of the D&M ISS Model in the context of this study embodies a sense of a diagnostic and interpretive device for healthcare professionals in different healthcare contexts. While the D&M ISS Model was operationalized within the Saudi public healthcare sector, it offers a transferable analytical lens that invites healthcare professionals to embark on experiential insights to tailor chatbot success strategies to their unique environments. In practice, this means that the enactment of IS success in intelligent healthcare systems is contextually constructed, reflecting the interplay of technical, organizational, and human factors. In this sense, the study sheds light on the contextual conditions under which information quality, system quality, service quality, and trust interact to influence user satisfaction, system use, and net benefits.

### 6.2. Limitation

Several limitations were acknowledged in this study. First, the use of convenience sampling may have introduced selection bias. This non-probability design supports feasibility in an otherwise hard-to-reach population, but it also constrains external validity and limits generalization beyond the study sample [125,126]. Second, the sample was heavily male-dominated, with women underrepresented. Because prior research suggests that gender can shape technology-related perceptions and adoption behavior, the observed relationships may not be identical in a more gender-balanced sample [146,147]. We therefore avoid claiming that the results would remain unchanged under balanced gender parity. Instead, the gender imbalance should be viewed as a boundary condition on the interpretation of the findings. Third, although the sample size was adequate for PLS-SEM, statistical sufficiency does not resolve representativeness concerns. Future research should replicate the model using stratified sampling or a more balanced design across gender groups, and it may also be useful to test for measurement or structural differences across male and female respondents [125]. Such work would help determine whether the present model is stable across subgroups and whether the effects observed here generalize more broadly. Finally, although the measurement and structural models met the main evaluation criteria, some VIF values exceeded the more conservative threshold of 4.0, suggesting a degree of multicollinearity among predictors that may affect the precision of the estimated relationships. While this does not necessarily invalidate the structural results, it indicates that the model should be interpreted with caution. Future research should therefore further examine this issue using larger and more diverse samples, and, where appropriate, apply additional diagnostic tests or alternative model specifications to assess the stability of the path estimates.

## Figures and Tables

**Table 1:** Measurement constructs, item codes, item statements, and original literature sources from which each item was adopted.

Code.	Measurements Items	Sources
System Quality		
SYQ1	I find it easy to use the MOH chatbot service to find what I want	[63]
SYQ2	I find the MOH chatbot service flexible to interact with	
SYQ3	I think using the MOH chatbot service is enjoyable	
SYQ4	This MOH chatbot service is user-friendly	
Information Quality		
INQ1	MOH Chatbot service provides up-to-date information	[63,88,105,113]
INQ2	MOH Chatbot service provides accurate information	
INQ3	The information provided by MOH Chatbot service is reliable	
INQ4	The information provided by MOH Chatbot service is understandable.	
Service Quality		
SEQ1	This MOH chatbot service provides me with an instant response	[63,64]
SEQ2	This MOH chatbot service gives me the personalized attention	
SEQ3	This MOH chatbot service provides me with the exact appropriate solution to my requirements	
SEQ4	The MOH chatbot service was always available whenever I needed it	
Trust		
TRU1	I believe that this MOH chatbot service is trustworthy	[64]
TRU2	(Has high integrity) I don’t doubt the honesty of the information provided by this MOH chatbot service	
TRU3	(Keeps my best interests in mind) I feel that MOH acts in citizen’s best interest	
TRU4	(Does the right job) Overall, I trust this MOH chatbot service	
Intention to use		
USE1	I will always try to use MOH chatbot service when I have the need	[64]
USE2	You are going to use MOH chatbot service in the future	
USE3	You are dependent on the MOH chatbot service	
USE4	You will often use the MOH chatbot service in the future	
User Satisfaction		
USR1	You are satisfied with the MOH chatbot service	[63,64]
USR2	The MOH chatbot service has met my expectations	
USR3	My decision to use the MOH chatbot service was a wise one	
USR4	This MOH’s chatbot efficiently fulfilled my needs	
Net Benefits		
NET1	MOH chatbot service helps me identify problems	[64]
NET2	The MOH chatbot service saves my time	
NET3	I think using the MOH chatbot service is useful to me	
NET4	MOH chatbot service helps me make higher-quality decisions	

**Table 2:** Psychometric indices and factor loadings for the measurement model constructs.

Construct/Items	FL	CR	α	AVE
Information Quality		0.938	0.912	0.791
INQ1	0.903			
INQ2	0.891			
INQ3	0.891			
INQ4	0.873			
Net Benefit		0.958	0.942	0.852
NET1	0.920			
NET2	0.921			
NET3	0.925			
NET4	0.927			
Satisfaction		0.957	0.940	0.847
USR1	0.926			
USR2	0.916			
USR3	0.923			
USR4	0.918			
Service Quality		0.924	0.891	0.754
SEQ1	0.848			
SEQ2	0.866			
SEQ3	0.902			
SEQ4	0.856			
System Quality		0.919	0.882	0.739
SYQ1	0.863			
SYQ2	0.892			
SYQ3	0.826			
SYQ4	0.857			
Trust		0.917	0.941	0.801
TRU1	0.915			
TRU2	0.879			
TRU3	0.870			
TRU4	0.914			
Use		0.954	0.935	0.838
USE1	0.918			
USE2	0.928			
USE3	0.898			
USE4	0.918			

**Table 3:** Matrix of Fornell–Larcker criteria showing the discriminant validity between latent variables in the measurement model.

Fornell–Larcker Criterion	Information Quality	Net Benefit	User Satisfaction	Service Quality	System Quality	Trust	Use
Information Quality	0.890						
Net Benefit	0.825	0.923					
Satisfaction	0.819	0.914	0.921				
Service Quality	0.795	0.813	0.830	0.868			
System Quality	0.806	0.769	0.763	0.731	0.860		
Trust	0.807	0.812	0.815	0.793	0.698	0.895	
Use	0.801	0.881	0.906	0.803	0.711	0.815	0.915

**Table 4:** Matrix of Heterotrait–Monotrait Ratio (HTMT) criteria showing the discriminant validity between latent variables in the measurement model.

HTMT Criterion	Information Quality	Net Benefit	User Satisfaction	Service Quality	System Quality	Trust	Use
Information Quality							
Net Benefit	0.889						
Satisfaction	0.884	0.970					
Service Quality	0.884	0.886	0.904				
System Quality	0.899	0.843	0.836	0.825			
Trust	0.881	0.872	0.874	0.879	0.775		
Use	0.867	0.938	0.966	0.878	0.780	0.875	

**Table 5:** Effect size estimates and structural path coefficients summarizing direct and total effects of model constructs on key outcome variables.

Path	H	Direct/Total	Path Coeff	t-Value	*p*-Values	F^2^	Effect
Information Quality -> Use	H1	Direct	0.237	2.923	0.003 **	0.049	Small
Information Quality -> Satisfaction	H2	Direct	0.089	1.191	0.234	0.013	Small
System Quality -> Use	H3	Direct	0.063	1.060	0.289	0.005	No effect
System Quality -> Satisfaction	H4	Direct	0.139	2.373	0.018 *	0.047	Moderate
Service Quality -> Use	H5	Direct	0.294	3.339	0.001 **	0.101	Moderate
Service Quality -> Satisfaction	H6	Direct	0.187	3.215	0.001 **	0.072	Moderate
Use -> Satisfaction	H7	Direct	0.585	9.567	0.000 ***	0.708	Large
Use -> Net Benefit	H8	Direct	0.300	3.626	0.000 ***	0.109	Moderate
Satisfaction -> Net Benefit	H9	Direct	0.642	8.140	0.000 ***	0.497	Large
Trust -> Use	H10	Direct	0.346	4.221	0.000 ***	0.139	Moderate
Information Quality -> Net Benefit	-	Total	0.218	2.956	0.003 **	-	-
Satisfaction -> Net Benefit	-	Total	0.642	8.140	0.000 ***	-	-
Service Quality -> Net Benefit	-	Total	0.319	4.240	0.000 ***	-	-
System Quality -> Net Benefit	-	Total	0.132	2.088	0.037 *	-	-
Trust -> Net Benefit	-	Total	0.234	3.924	0.000 ***	-	-
Trust -> Satisfaction	-	Total	0.202	3.760	0.000 ***	-	-

* *p* < 0.1; ** *p* < 0.01; *** *p* < 0.001.

**Table 6:** Model explanatory power and multicollinearity diagnostics, including R^2^ and Q^2^ values for endogenous constructs and VIF and tolerance statistics for predictor variables.

Constructs	R^2^	Q^2^	VIF	Tolerance
Net Benefit	0.851	0.720	6.711	0.149
User Satisfaction	0.864	0.726	7.353	0.136
Use	0.753	0.619	4.049	0.247

## Data Availability

The anonymized dataset used in this study is available upon reasonable request from qualified researchers, subject to approval by the corresponding author, due to data privacy and confidentiality. Justification for access, including a detailed research proposal, should be provided.

## Abbreviations

The following abbreviations are used in this manuscript:

AI	Artificial Intelligence
AVE	Average Variance Extracted
CB-SEM	Covariance-Based Structural Equation Modeling
CR	Composite Reliability
D&M ISS Model	DeLone and McLean Information Systems Success Model
FL	Factor Loading
INQ	Information Quality
MOH	Ministry of Health
NET	Net Benefit
PLS-SEM	Partial Least Squares Structural Equation Modeling
Q^2^	Predictive Relevance (Stone-Geisser’s Q^2^)
R^2^	Coefficient of Determination
SEM	Structural Equation Modeling
SEQ	system quality
SERVQUAL	Service Quality Model
SYQ	System Quality
TAM	Technology Acceptance Model
TRU	Trust
USE	Intention to Use
USR	User Satisfaction
UTAUT	Unified Theory of Acceptance and Use of Technology
VIF	Variance Inflation Factor
